# Characterization of the Interaction of Full-Length HIV-1 Vif Protein with its Key Regulator CBFβ and CRL5 E3 Ubiquitin Ligase Components

**DOI:** 10.1371/journal.pone.0033495

**Published:** 2012-03-30

**Authors:** Xiaohong Zhou, Sean L. Evans, Xue Han, Yayan Liu, Xiao-Fang Yu

**Affiliations:** 1 Institute of Virology and AIDS Research, First Affiliated Hospital of Jilin University, Jilin, People's Republic of China; 2 Department of Molecular Microbiology and Immunology, Johns Hopkins Bloomberg School of Public Health, Baltimore, Maryland, United States of America; New York Blood Center, United States of America

## Abstract

Human immunodeficiency virus-1 (HIV-1) viral infectivity factor (Vif) is essential for viral replication because of its ability to eliminate the host's antiviral response to HIV-1 that is mediated by the APOBEC3 family of cellular cytidine deaminases. Vif targets these proteins, including APOBEC3G, for polyubiquitination and subsequent proteasome-mediated degradation via the formation of a Cullin5-ElonginB/C-based E3 ubiquitin ligase. Determining how the cellular components of this E3 ligase complex interact with Vif is critical to the intelligent design of new antiviral drugs. However, structural studies of Vif, both alone and in complex with cellular partners, have been hampered by an inability to express soluble full-length Vif protein. Here we demonstrate that a newly identified host regulator of Vif, core-binding factor-beta (CBFβ), interacts directly with Vif, including various isoforms and a truncated form of this regulator. In addition, carboxyl-terminal truncations of Vif lacking the BC-box and cullin box motifs were sufficient for CBFβ interaction. Furthermore, association of Vif with CBFβ, alone or in combination with Elongin B/C (EloB/C), greatly increased the solubility of full-length Vif. Finally, a stable complex containing Vif-CBFβ-EloB/C was purified in large quantity and shown to bind purified Cullin5 (Cul5). This efficient strategy for purifying Vif-Cul5-CBFβ-EloB/C complexes will facilitate future structural and biochemical studies of Vif function and may provide the basis for useful screening approaches for identifying novel anti-HIV drug candidates.

## Introduction

Virion (or viral) infectivity factor (Vif), a 23-kDa accessory protein of human immunodeficiency virus type 1 (HIV-1) and many related lentiviruses, is essential for viral replication. Vif inactivates the antiretroviral activity of the host APOBEC3 cytidine deaminases, including APOBEC3G (A3G) and A3F [1], [2], [3], [4], [5], [6]. A3G [7] and related human APOBEC3 proteins are potent inhibitors of HIV-1 in the absence of viral Vif. A3G can be packaged into HIV-1 particles through the nucleocapsid RNA-binding domain (NC) of viral Gag [8], [9], [10], [11], [12], [13], [14], [15], [16], along with a contribution from viral or cellular RNAs [8], [9], [10], [11], [12], [13], [14], [15], [16], [17], [18]. A3G viral packaging leads to the induction of C-to-U mutations in the minus-strand viral DNA during reverse transcription within newly infected cells [19], [20], [21], [22], [23], [24], [25]. In addition, virion-packaged APOBEC3 proteins can reduce the accumulation of viral DNA [26], [27], [28], [29], [30], [31], [32], [33] and the formation of proviral DNA [23], [28], [29], [34].

Vif induces polyubiquitination and degradation of multiple APOBEC3 molecules [35], [36], [37], [38], [39], [40], [41], [42]. Specifically, Vif acts as a substrate receptor for specific APOBEC3 proteins, while also recruiting a cullin5-RING (CRL5) ubiquitin ligase complex composed of Cul5, ElonginB (EloB), ElonginC (EloC), and a RING-box protein [35] through a highly conserved virus-specific BC-box motif [36], [43] and a HCCH motif [44], [45], [46], [47]. Various Vif motifs have been found to participate in the interaction of HIV-1 Vif with diverse substrates [6], [38], [48], [49], [50], [51], [52], [53], [54], [55], [56], [57], [58].

To determine how Vif hijacks the CRL5 E3 ligase in order to degrade the antiviral proteins A3G and A3F, researchers have sought to characterize Vif-E3 ligase-related complexes, such as EloB/C with a Vif C-terminal fragment (residues 139–176) [59], synthetic Vif C-terminal domains [60], [61], [62], and EloB/C-Vif-Cul5 interactions [63], [64]. These studies have identified important motifs responsible for the interaction between Vif, EloB/C and Cul5.

However, structural and functional analyses of full-length Vif continue to be limited by difficulty in obtaining suitable quantities of soluble full-length Vif protein [65], [66], [67], [68]. In an attempt to overcome this limitation, a denaturing/refolding method has been developed for purifying soluble recombinant Vif [65], [68], [69], [70]. Although this approach produced large quantities of full-length protein, the protein formed high molecular weight aggregates in solution [65], [68], [69], [70]. Vif's tendency to aggregate and become insoluble has limited its structural characterization and functional analysis [63].

Co-expression of binding partners has been shown to improve the solubility and stability of various proteins [71]. Here, we report that co-expression of Vif with EloB/C and CBFβ, a newly identified regulator of HIV-1 Vif function [72], [73], [74], [75], can greatly improve the solubility of full-length Vif. We also demonstrate that C-terminal truncated Vif mutants of up to 140 amino acids can still interact with CBFβ. Purified amino-terminal domain of Cul5 (residues 1–393) readily interacts with this complex. Vif-CBFβ-EloB/C-Cul5 complexes purified by our strategy were not prone to aggregate and can therefore facilitate future structural and biochemical studies of Vif function.

## Materials and Methods

### Cloning, expression, and purification

**Figure 1:**
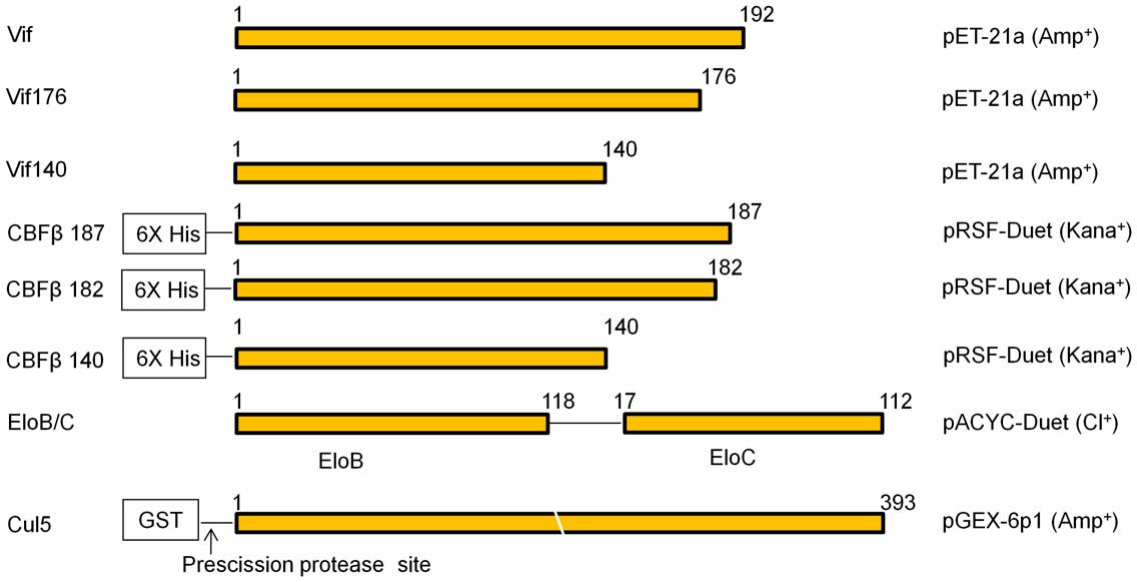
Constructs used in this study. The vectors and their antibiotic resistance are given, followed by a diagram of the construct: Amp^+^, ampicillin; Kana^+^, kanamycin; and Cl^+^, chloramphenicol. Elongins B and C (EloB/C) are in a single vector (pACYC-Duet) regulated by dual promoters. The Precission protease sequence site in glutathione S-transferase (GST) and Cul5 are indicated.

Full-length Vif192 in the pET21 vector was a gift from Drs. Rahul M. Kohli and James T. Stivers. Truncated Vif176 and Vif140 were cloned into pET21 vector. Elongin B and Elongin C (residues 17 to 112) in the pACYC-Duet plasmid were a gift from Alex Bullock (University of Oxford, Oxford, United Kingdom). Mouse CBFβ (residues 1–187) cDNA were a gift from Nancy A. Speck (University of Pennsylvania). CBFβ isoform 1 (residues 1–187) from mouse, CBFβ isoform 2 (residues 1–182) and truncated CBFβ (residues 1–140) from human were cloned into pRSF-Duet. For expression, the plasmids were transformed into *Escherichia coli* BL21(DE3) cells. The constructs used in this study are summarized in Fig. 1. The proteins were over-expressed overnight at 16°C by induction with 0.1 mM isopropyl-D-thiogalactopyranoside (IPTG). Harvested cells were lysed in 20 mM Tris-HCl, pH 8.0, with 150 mM NaCl and then clarified by sonication and centrifugation at 13,000 g for 30 min. For solubility analysis, the supernatant was removed and the pellet resuspended to the original volume. For nickel affinity purification, the supernatant was transferred to Ni-NTA beads (Invitrogen), and the flowthrough was loaded onto Ni-NTA beads for two more passages. After washing with 20 mM Tris-HCl, pH 8.0, with 150 mM NaCl and 40 mM imidazole, the protein complex was eluted with 20 mM Tris-HCl, pH 8.0, with 150 mM NaCl and 400 mM imidazole. Gel filtration and anion exchange were utilized to remove trace contamination. Cul5-NTD (residues 1 to 393 with two point mutations, V341R and L345D) in the pGEX-6p-1 vector was expressed in *E. coli* BL21(DE3) cells overnight at 16°C by induction with 0.1 mM IPTG. Harvested cells were lysed by sonication in 20 mM Tris-HCl, pH 8.0, with 150 mM NaCl, then clarified by centrifugation at 13,000 g for 30 min. The supernatant was transferred to glutathione-Sepharose 4B beads (GE Healthcare) for glutathione S-transferase (GST) affinity chromatography. The GST tag was then removed using Prescission protease. Gel filtration chromatography was utilized for further purification.

### Gel filtration chromatography

Each Vif complex and Cul5 sample was concentrated to 300 µl and loaded onto a Superdex 200 (10/300 GL) column (GE Healthcare) with a 500-µl loop and run at a flow rate of 0.3 ml per min; the column was calibrated using vitamin B12 (1,370 Da), myoglobin (17,000 Da), ovalbumin (44,000 Da), gamma globulin (158,000 Da), and thyroglobulin (670,000 Da) as standards. The gel filtration buffer for Vif-CBFβ was composed of 20 mM Tris-HCl pH 8.0, with 150 mM NaCl and 10% glycerol. The gel filtration buffer for Vif-CBFβ-EloB/C, Vif- CBFβ-EloB/C-Cul5, and Cul5 was 20 mM Tris-HCl, pH 8.0, with150 mM NaCl.

### Pull-down analysis of the Vif-CBFβ interaction

For pull-down experiments analyzing the interactions between Vif and CBFβ, supernatant was incubated on Ni-NTA agarose for 30 min at 4°C. After incubation, the reaction mixtures were washed 10 times with 1 ml lysis buffer. The samples were then analyzed by SDS-PAGE and visualized with Coomassie staining or by immunblotting with specific antibodies.

### Immunoblot analysis

Proteins were separated by SDS-PAGE, then transferred to nitrocellulose membranes (Bio-Rad). After blocking with PBS-buffered saline-Tween 20 containing 5% BSA for 1 h at room temperature, membranes were incubated with a specific antibody overnight at 4°C. After three washes with PBS-buffered saline-Tween 20, the membranes were stained with an alkaline phosphatase-conjugated secondary antibody (1∶5,000, Sigma) for 1 h at room temperature. After three washes with PBS-buffered saline-Tween 20, the membranes were reacted with 5-bromo-4-chloro-3′-indolylphosphate (BCIP) and nitro-blue tetrazolium (NBT) substrate (Sigma). The antibodies used in this study were specific for: Vif (the AIDS Research Reagents Program, Catalog #2221), CBFβ (Abcam, Catalog ab11921), EloB (Santa Cruz Biotechnology, Inc, Catalog sc-11447), EloC (BD Transduction Laboratories, Catalog 610760), Alkaline Phosphatase-conjugated secondary mouse and rabbit (Jackson Immunoresearch, Catalog 111-055-045 and 115-055-003).

## Results

### CBFβ co-expression improves the solubility of Vif

**Figure 2:**
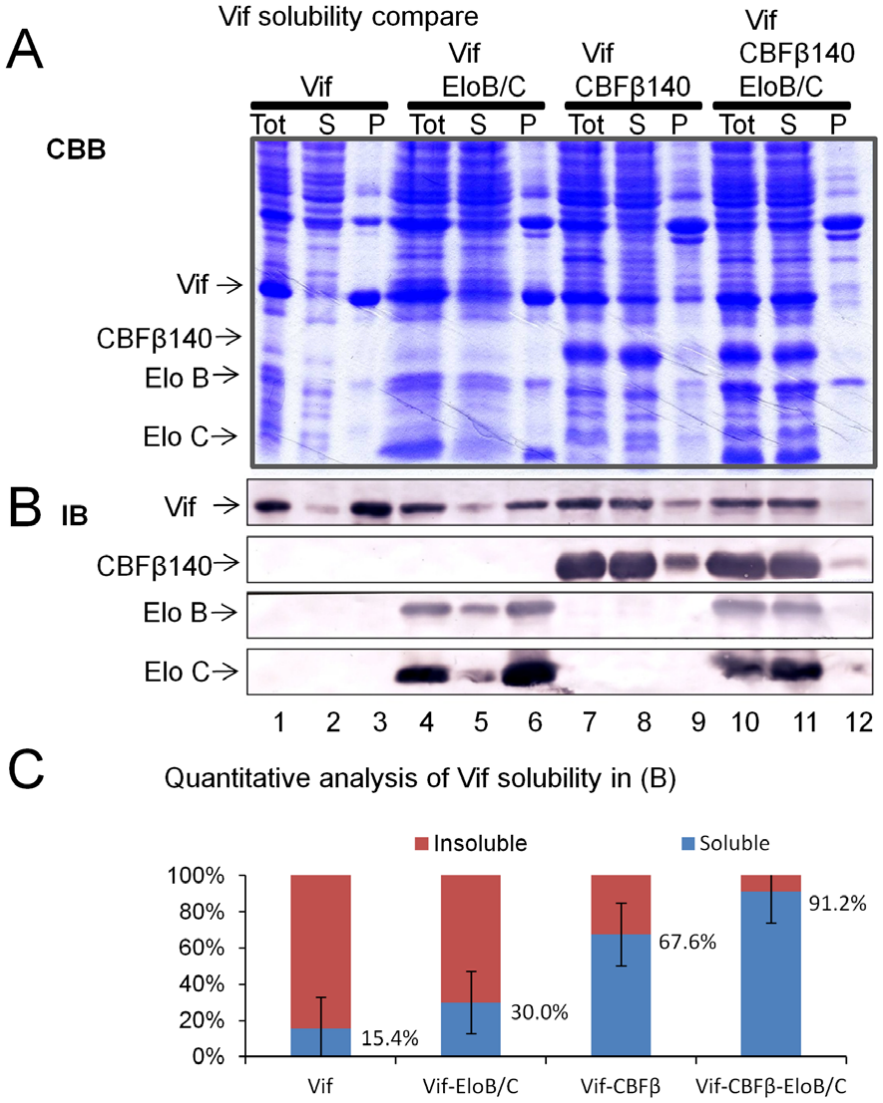
Soluble Vif protein was obtained by co-expression with CBFβ and EloB/C. Vif was untagged, while CBFβ was tagged with 6X His residues at the N-terminus. (A) Solubility of Vif alone and of co-expressed Vif-EloB/C, Vif-CBFβ, and Vif-CBFβ-EloB/C. Tot, total lysate; S, supernatant; P, pellet; CBB, Coomassie staining. (B) Fractions in (A) were checked by Immunoblotting (IB) using protein-specific antibodies. (C) Quantification of Vif protein by immunoblotting in (B).

To identify strategies that could result in the expression of large quantities of soluble full-length Vif recombinant proteins, we constructed various prokaryotic expression vectors for HIV-1 Vif and its co-factors (Fig. 1). Recombinant Vif protein (residues 1–192) was efficiently expressed in *E. coli* BL21(DE3) but remained predominantly insoluble as indicated by Coomassie staining (Fig. 2A, lanes 1–3). The Vif protein was also identified by immunoblotting using a Vif-specific antibody (Fig. 2B, lanes 1–3). Co-expression with EloB/C improved the solubility of Vif, but only to a limited extent (Fig. 2A and B, lanes 4–6). When Vif was co-expressed with CBFβ140-His (residues 1–140 of CBFβ with six histidine residues at the N-terminus), the solubility of Vif improved significantly (Fig. 2A and B, lanes 7–9). Approximately 67% of the total Vif protein became soluble in the presence of CBFβ140-His (Fig. 2C). Expressing CBFβ and EloB/C together further enhanced the solubility of Vif (Fig. 2A and B, lanes 10–12). When Vif was co-expressed with CBFβ and EloB/C, >90% of the Vif proteins became soluble (Fig. 2C).

### CBFβ interacts with Vif

**Figure 3:**
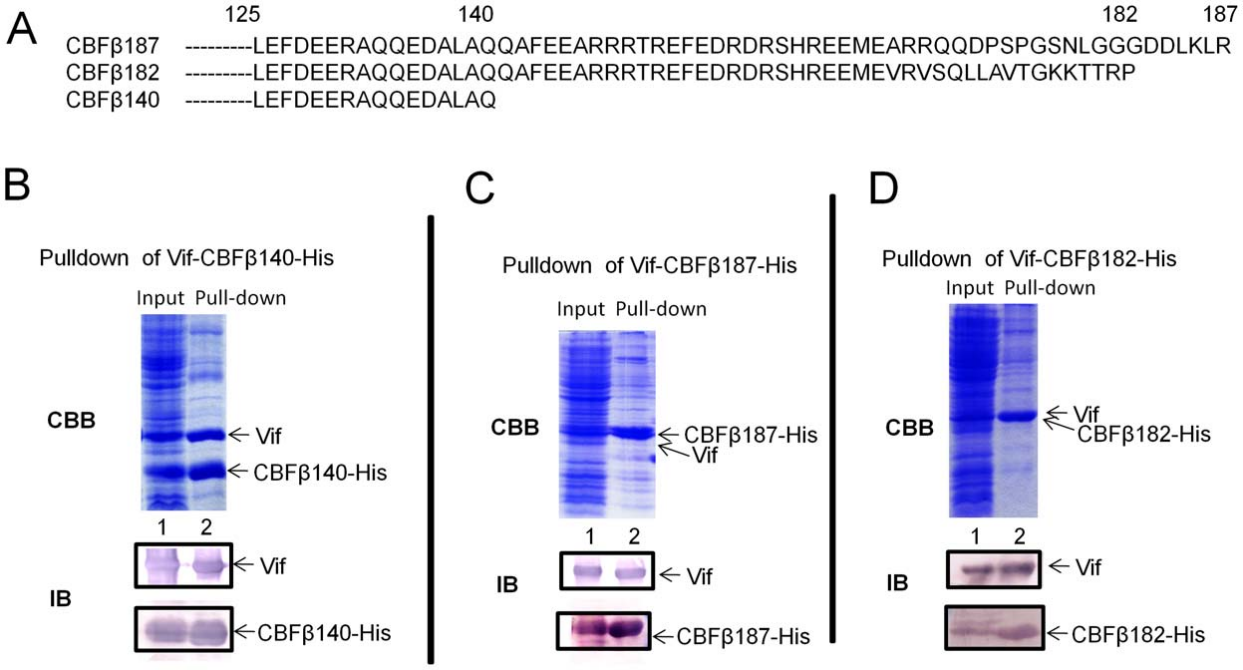
CBFβ interacts with Vif via its N-terminal domain. (A) Sequence alignment of CBFβ isoforms (CBFβ187 or CBFβ182) and truncated CBFβ140. The N-terminus is omitted. (B), (C), (D) Vif was pulled down by His-tagged CBFβ: CBFβ140 (B), CBFβ187 (C), or CBFβ182 (D). Supernatant from the cell lysates was used as the input sample. Both input and pull-down samples were checked by SDS-PAGE, followed by Coomassie staining or immunoblotting.

The ability of CBFβ140-His to increase the solubility of Vif suggests that there is an interaction between Vif and CBFβ140-His. To determine whether Vif and CBFβ could interact directly, we attempted to co-precipitate Vif with CBFβ140-His and found that Vif in the soluble fraction could be efficiently pulled down by the CBFβ140-His on a nickel column (Fig. 3B, lane 2). The presence of Vif and CBFβ140-His in the soluble input fraction and the co-precipitated samples was confirmed by immunoblotting using a Vif- or CBFβ-specific antibody (Fig. 3B).

There are two major CBFβ isoforms that are highly conserved in mammals ([76], [77]: Isoform1 has 182 amino acids, while isoform 2 has a 187 amino acid sequence that is generated by alternative splicing. The two isoforms differ in the last 22 amino acids (Fig. 3A). Human and mouse CBFβ differ by two amino acids (42 A/T and 117 Q/H). Next, we asked whether the natural isoforms of CBFβ could interact with Vif and found that an interaction did indeed occur between HIV-1 Vif and isoform 1 CBFβ182 (Fig. 3C) as well as isoform 2 CBFβ187 (Fig. 3D) in co-precipitation experiments. To our knowledge, this is the first reported evidence of a direct interaction between HIV-1 Vif and various forms of CBFβ, *in vitro*. Our data also indicate that amino acids 1–140 of CBFβ are sufficient for HIV-1 Vif binding.

### Purified Vif-CBFβ-EloB/C proteins form a stable monomeric complex

**Figure 4:**
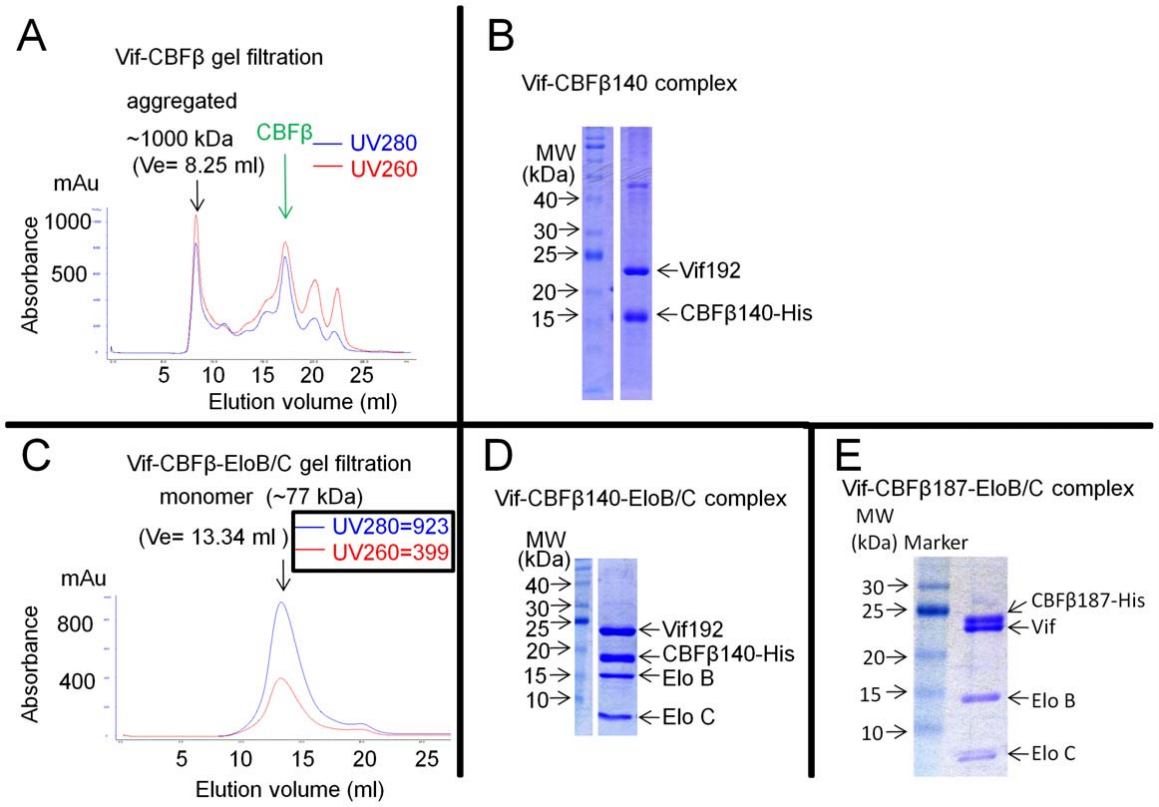
Vif-CBFβ-EloB/C forms a homogenous monomeric complex. (A) Gel filtration profile of Vif-CBFβ complexes on Superdex 200 (GE Healthcare). The elution volume (Ve) and corresponding molecular size (MW, calculated using protein standards) are indicated by arrows. (B) SDS-PAGE and Coomassie staining of Vif-CBFβ complexes from the peak fractions in (A). (C) Gel filtration profile of Vif-CBFβ-Elo B/C complexes on Superdex 200. The theoretical calculated molecular size of the monomeric Vif-CBFβ-EloB/C was ∼65 kDa. (D) SDS-PAGE and Coomassie staining of Vif-CBFβ-EloB/C complexes from the peak fraction in (C). (E) Purified Vif-CBFβ187-EloB/C complexes.

Soluble Vif and CBFβ140 complexes were purified by nickel affinity chromatography and analyzed by gel filtration using a Superdex200 10/300 GL size exclusion column. Gel filtration analysis (Fig. 4A) suggested that Vif and CBFβ140 formed a large aggregated complex of approximately 1000 kDa. Protein analysis by Coomassie staining of the peak fraction after separation by SDS-PAGE suggested a 1∶1 ratio of Vif∶CBFβ140 (Fig. 4B). Full length or truncated CBFβ were monomeric in solution [78]. This observation supports previous findings that Vif directly interacts with CBFβ [72]. Gel filtration analysis of purified Vif-CBFβ140-EloB/C revealed that the complex formed a homogeneous complex of ∼65–75 kDa (Fig. 4C). Protein analysis by Coomassie staining of the peak fraction indicated a 1∶1∶1∶1 ratio of Vif∶CBFβ140∶EloB∶EloC (Fig. 4D) or Vif∶CBFβ187∶EloB∶EloC (Fig. 4E). The calculated molecular weight of the monomeric Vif-CBFβ140-EloB/C complex (∼65 kDa) was in close agreement with our gel filtration results (∼75 kDa) suggesting that Vif-CBFβ-EloB/C complex is a monomeric complex in solution.

**Figure 5:**
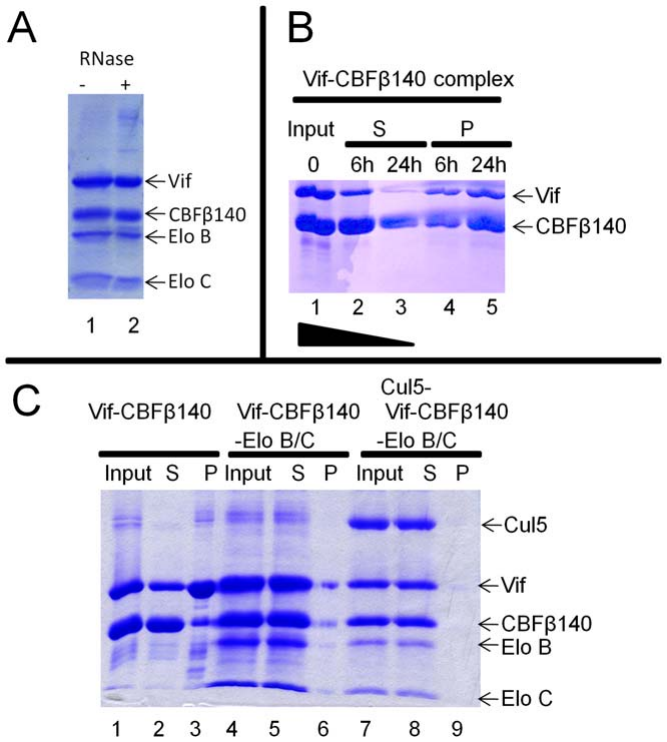
Vif-CBFβ-EloB/C is an RNA-independent stable complex in solution. (A) Vif-CBFβ-EloB/C was not dependent on RNA. The purified complex (2 mg/ml, 100 µl) was incubated with 2 µl RNase Mix (RNase A/T1 Mix, Catalog EN055, Fermentas) at 37°C for 4 h according to the manufacturer's protocol, followed by buffer exchange to remove the EDTA. The complex then was analyzed by His-tag affinity pull-down. (B) The Vif-CBFβ complex is not stable. Purified Vif-CBFβ complexes were concentrated to 4 mg/ml and, after clarification at 13,000 g for 10 min, the supernatants were stored at 4°C (Input). Samples were then removed at different times (0 h, 6 h, 24 h), and after clarification at 13,000 g for 10 min, the supernatants (S) were removed and the pellets (P) resuspended to the original volume and checked by SDS-PAGE. (C) Purified Vif complexes were concentrated to 5 mg/ml (Input) and stored at 4°C overnight (∼16 h). The supernatants (S) were removed after clarification at 13,000 g for 10 min, and the pellets (P) were resuspended to the original volume, then checked by SDS-PAGE.

The stability of the purified Vif-CBFβ140 complexes was low: at 4°C, the complexes precipitated after only a few hours (Fig. 5B). After 16 h at 4°C, >50% of the Vif protein precipitated (Fig. 5C, lanes 1–3). More Vif protein than CBFβ140 protein appeared in the precipitates, although the initial ratio of Vif and CBFβ was about 1∶1 (Fig. 5C, lane 1).In contrast, the Vif-CBFβ140-EloB/C complexes were more stable (Fig. 5A, lanes 4–6): only a trace amount of Vif precipitated after 16 h at 4°C.

Previous studies have suggested that HIV-1 Vif can bind RNA [79], [80], [81], [82]. We found that the Vif-CBFβ140-EloB/C complexes were resistant to RNase treatment (Fig. 5A). Purified Vif-CBFβ140-EloB/C complexes were untreated or treated with 40 µg/ml of RNase A and 20 U/ml RNase T1 at 37°C for 4 h. After buffer exchange, the treated samples were purified using nickel columns. RNase treatment did not affect the co-purification of Vif, EloB, and EloC with CBFβ140-His (Fig. 5A, lane 2) when compared to the untreated sample (lane 1). These data suggest that the Vif-CBFβ-EloB/C complexes are not RNA-dependent. The OD280/260 ratio in the peak fraction of the Vif-CBFβ140 -EloB/C complexes (Fig. 4C) also argued against the presence of RNA.

### Interaction of CBFβ with Vif truncation mutants

**Figure 6:**
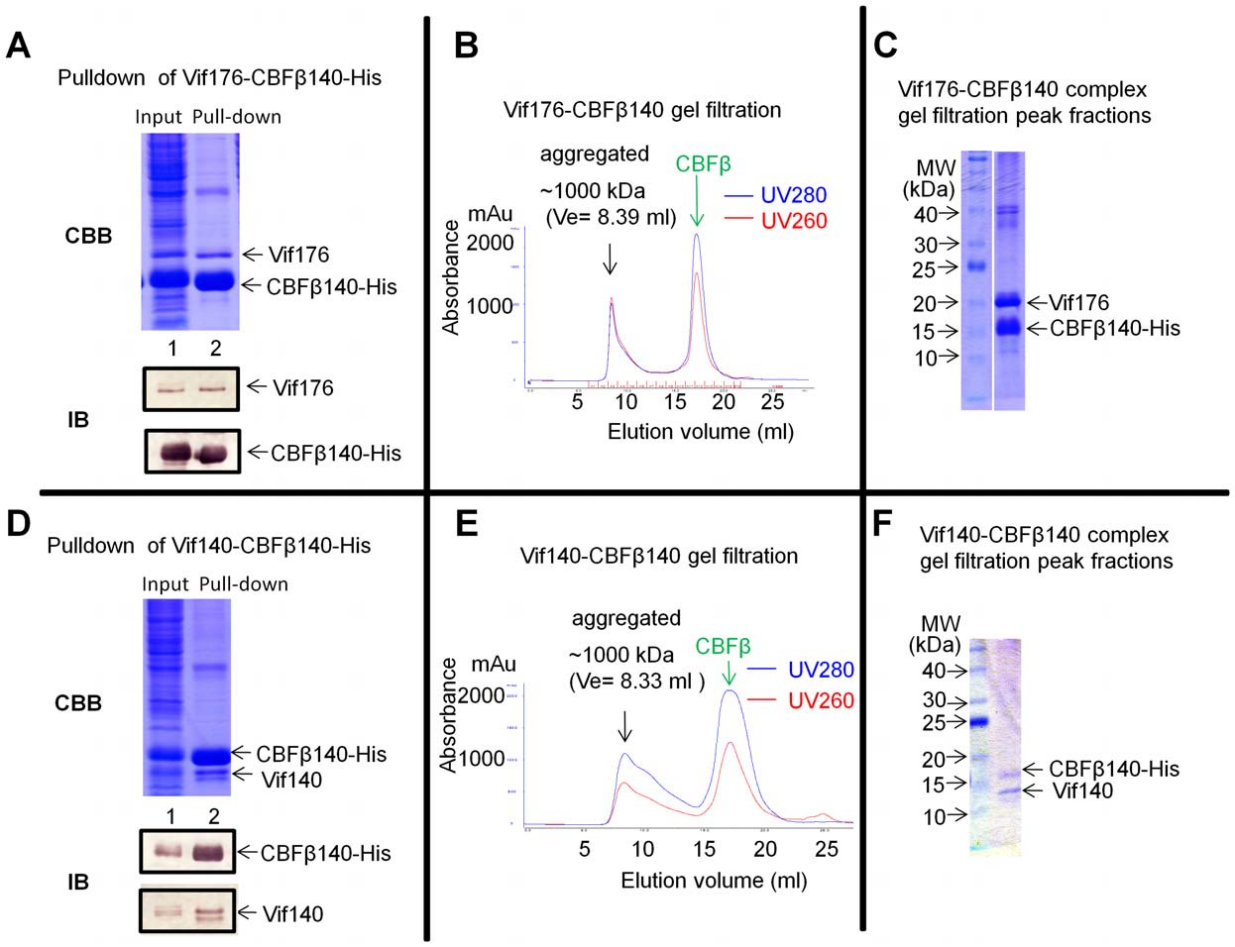
Vif140 and Vif176 bind to CBFβ and form aggregated complexes. (A) and (D), Vif was co-expressed with CBFβ. The supernatant from the cell lysates was used as the input sample. Both input and pull-down samples were checked by SDS-PAGE, followed by Coomassie staining or blotting. (B) and (E), The purified Vif- CBFβ140 complexes were checked by gel filtration on a Superdex200 10/300 column. (C) and (F), The peak factions were analyzed by SDS-PAGE and Coomassie staining.

We next asked which region of Vif was required for the interaction between Vif and CBFβ. Two truncated Vif mutants spanning residues 1–176 and 1–140 were constructed and co-expressed with CBFβ140-His. Truncated Vif in the soluble fractions was analyzed by co-precipitation with CBFβ140-His using nickel beads. SDS-PAGE and Coomassie staining indicated that both truncated Vif176 (Fig. 6A) and Vif140 (Fig. 6D) co-precipitated with CBFB140-His; this finding was confirmed by immunoblotting with a Vif- or CBFβ-specific antibody (Fig. 6A and D).The pulldown fractions were further analyzed by size exclusion. Both Vif176-CBFβ140 (Fig. 6B) and Vif140-CBFβ140 (Fig. 6E) formed large aggregates. Peak fractions were analyzed by SDS-PAGE followed by Coomassie staining. Both Vif176-CBFβ140 (Fig. 6C) and Vif140-CBFβ140 (Fig. 6F) showed a 1∶1 ratio of Vif∶CBFβ. These results suggested that N-terminal residues 1–140 of HIV-1 Vif are sufficient for CBFβ binding.

### Vif-CBFβ-EloB/C forms a complex with Cul5

**Figure 7:**
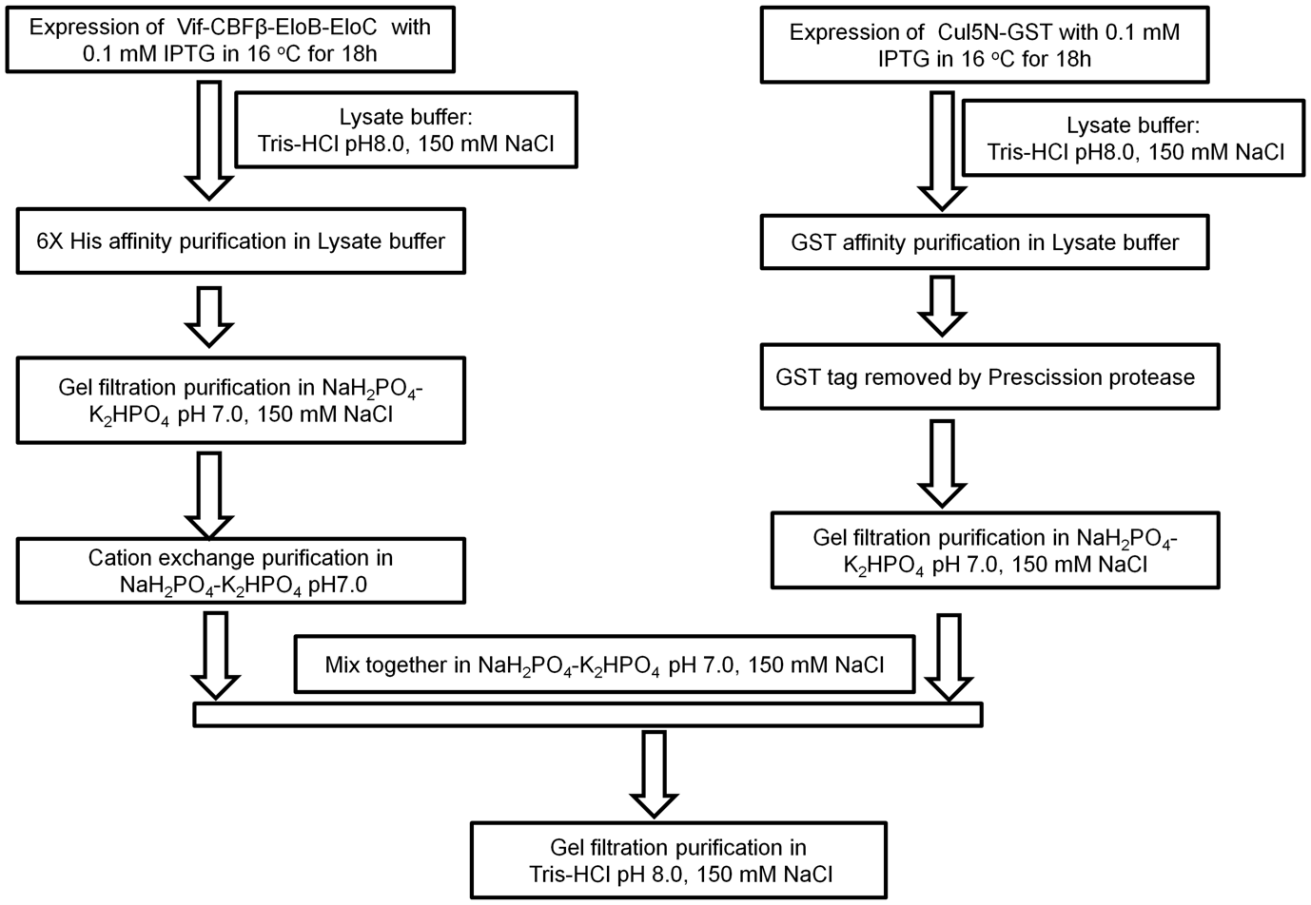
Purification strategy used in this study. IPTG, isopropyl-D-thiogalactopyranoside. Expression conditions are indicated, as are the buffers and methods used in each step.

**Figure 8:**
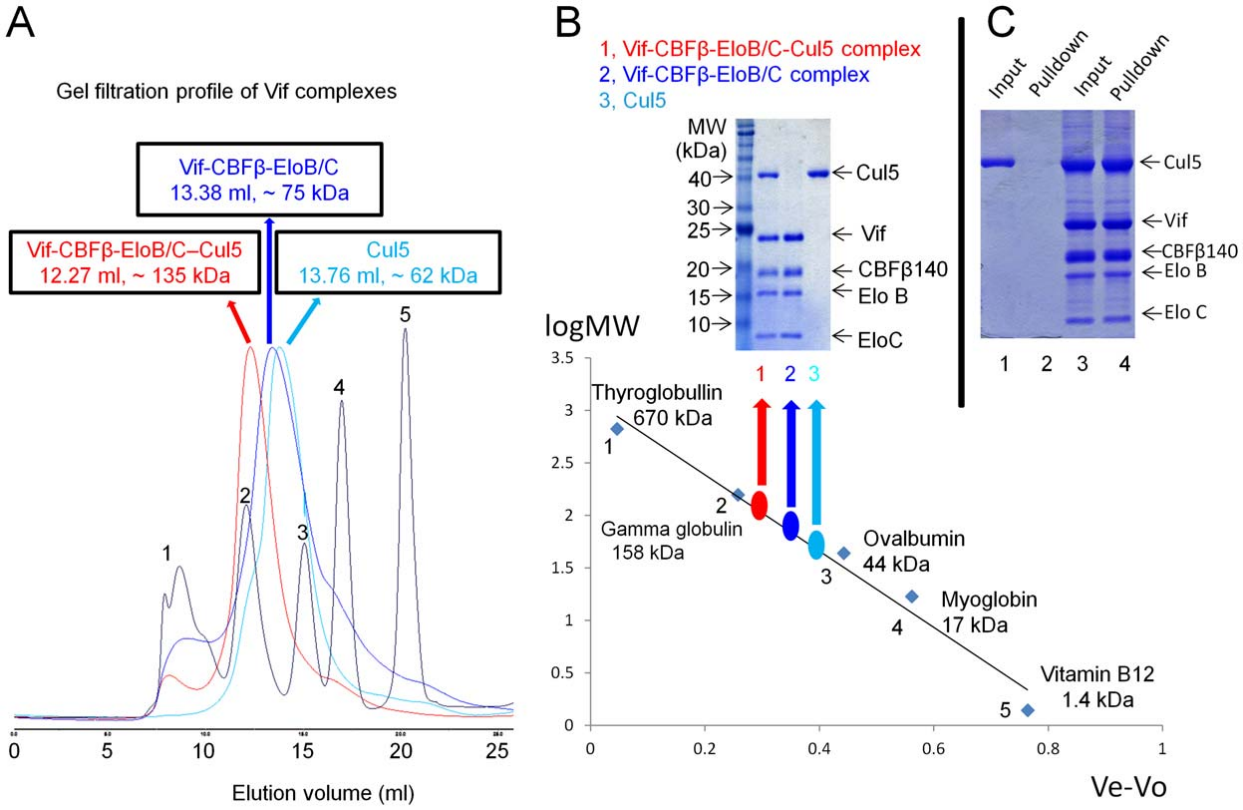
Purification of Vif-CBFβ-EloB/C complexes with Cul5. Cul 5, Cullin5; Elo B, Elongin B; Elo C, Elongin C. (A) Gel filtration profile of Vif-CBFβ-EloB/C complexes (blue line), Vif-CBFβ-EloB/C-Cul5 complexes (red line), and Cul5 (cyan line). The elution volume (Ve) in milliliters and molecular weights are indicated. The black line corresponds to the protein standards: 1, thyroglobulin (670,000 Da); 2, gamma globulin (158,000 Da); 3, ovalbumin (44,000 Da); 4, myoglobin (17,000 Da); 5, vitamin B12 (1,370 Da). (B) Peak fractions of protein complexes in (A) were checked by SDS-PAGE with Coomassie staining (upper panel): Cul5N-Vif-CBFβ-EloB/C complexes (lane 1), Vif-CBFβ-EloB/C complexes (lane 2), and Cul5N (lane 3). The molecular sizes of these complexes (as compared to the molecular standards) are shown in the lower panel: Vif-CBFβ-EloB/C complexes (blue), Vif-CBFβ-EloB/C-Cul5 complexes (red), and Cul5 (cyan). The standard proteins and their molecular weights are indicated. Ve, elution volume; Vo, void volume. (C) Affinity pull-down by nickel beads of the Vif-CBFβ-EloB/C-Cul5 complex peak fractions through the His-tagged CBFβ. Purified Cul5 protein was used as a control.

Because binding to Cul5 is essential for Vif-mediated ubiquitination and degradation of target proteins such as A3G and A3F, we next determined whether these purified Vif-CBFβ140-EloB/C complexes could interact with Cul5. Vif-CBFβ140-EloB/C complexes and Cul5 NTD were purified separately (Fig. 7). The purified Vif-CBFβ-EloB/C complexes were mixed with purified Cul5 protein and subsequently analyzed by gel filtration. As compared to Vif-CBFβ140-EloB/C (blue line) and Cul5 (cyan line), the mixture (red line) had an earlier elution peak (Fig. 8A). This result suggested that Vif-CBFβ140-EloB/C may form a complex with Cul5. SDS-PAGE analysis of the peak fractions suggested that Cul5 and Vif-CBFβ140-EloB/C formed a complex (Fig. 8B, upper panel). Molecular weight analysis by gel filtration (Fig. 8B, lower panel) indicated that the molecular size of the Vif-CBFβ140-EloB/C-Cul5 complex was approximately 135 kDa, equal to the sum of Cul5 (∼62 kDa) and Vif-CBFβ140-EloB/C (∼75 kDa). Further analysis using affinity pull-down via His-tagged CBFβ confirmed the formation of Cul5-Vif-CBFβ140-EloB/C complexes (Fig. 8C). These Vif-CBFβ140-EloB/C-Cul5 complexes were stable at 4°C over 16 h (Fig. 5C, lanes 7–9). The interaction between Cul5 and Vif-CBFβ-EloB/C suggests that Vif-CBFβ-EloB/C may be a functional complex, *in vivo*.

## Discussion

Human CBFβ has recently been identified as a critical regulator of HIV-1 Vif function [72], [73], [74], [75]. In the present study, we demonstrate that this host regulator directly interacts with Vif alone and in complex with E3 ligase components, *in vitro*. CBFβ is the non-DNA-binding subunit of a heterodimeric transcription factor, including RUNX family proteins [83], [84]. CBFβ regulates the folding and DNA-binding activity of RUNX partners, which play important roles in the development and differentiation of diverse cell types, including T lymphocytes and macrophages [83], [84]. We have recently reported that CBFβ is critical for Vif-induced A3G polyubiquitination and degradation [72]. Further clarification of the Vif-CBFβ-EloB/C-Cul5 interaction and complex assembly would provide key insights into how Vif recruits these E3 ligase components to degrade A3G/A3F.

Co-expression of HIV-1 Vif with CBFβ in the absence of all other human factors increased Vif solubility in *E. coli*. Soluble Vif could be co-precipitated with both His-tagged full length or truncated CBFβ (Fig. 3C, D, and E) In the absence of binding partners, previous research has suggested full length Vif appears to be unstructured and poorly soluble, *in vitro* [85]. Recently, Wolfe *et al.* were able to obtain soluble C-terminal domain fragments of Vif in complex with EloB/C and Cul5 [63]. Attempts at characterizing full length Vif in complex with EloB/C and Cul5 were unsuccessful, suggesting that the N-terminus was responsible for Vif's poor solubility, in the absence of N-terminal binding partners. We have shown that CBFβ binds the N-terminal region of Vif, specifically requiring hydrophobic interactions at amino acids W21 and W38 [72]. We hypothesize that the exposure of the N-terminal hydrophobic surface may contribute to Vif insolubilty when expressed alone. *In vivo*, CBFβ appears to be necessary for Vif-Cul5 binding, though CBFβ does not bind Cul5 directly [72], [73]. Thus, a possible role for CBFβ would be to stabilize Vif structure and promote the assembly of the Vif-Cul5 E3 ubiquitin ligase complex.

Vif and CBFβ co-fractionated in gel filtration analyses and appeared as a 1∶1 ratio complex. Isoforms 1 and 2 as well as a truncated form (amino acids 1–140) of CBFβ all interacted with HIV-1 Vif. Thus, most, if not all, of the Vif binding activity is preserved within the first 140 amino acids of CBFβ. Of note, C-terminal truncation of CBFβ up to amino acids 1–135 have been reported to bind and act in complex with RUNX family proteins [86]. In addition, we have confirmed that CBFβ binds to at least the first 140 amino acids of HIV-1 Vif. Thus, the known protein-binding domains in Vif, including the EloB/C binding BC-box, the cullin box containing the PPLP motif, are not essential for the Vif-CBFβ interaction. Vif forms homo-oligomers, and the PPLP motif has been suggested to be required for oligomerization [63], [70], [87], [88], [89], [90]. Since Vif140 still forms oligomers with CBFβ140, CBFβ182, and CBFβ187, our results suggest that regions in Vif in addition to PPLP may also participate in Vif oligomerization. This conclusion is consistent with the recent finding that the PPLP motif is not sufficient for Vif multimerization [64].

Biophysical and structural information for Vif has been limited as a result of its insolubility and strong tendency to oligomerize into high molecular weight aggregates. Of note, previous biochemical studies have employed full-length Vif protein obtained by the denaturing/refolding method [90] or have used truncated tagged protein [63]. Interestingly, when CBFβ and EloB/C were present, even untagged full-length Vif could be purified as a stable and soluble complex.

Association of Vif with CBFβ alone, and especially in combination with EloB/C, greatly increases the solubility of full-length Vif. We have shown that a stable complex containing Vif-CBFβ140-EloB/C can be purified in large quantities. This complex appeared to contain one subunit of each protein and did not dissociate upon RNase treatment. More importantly, the Vif-CBFβ140-EloB/C complexes we produced could interact with purified Cul5 and form stable Vif-CBFβ140-EloB/C-Cul5 complexes. This successful purification of monomeric Vif-E3 ligase complexes in high purity will greatly facilitate biochemical studies, structural determination, and functional analyses in this field.

Because CBFβ is a unique regulator of Vif's ability to hijack the cellular CRL5 E3 ligase, disrupting interactions within the Vif-CBFβ140-EloB/C-Cul5 complex represents an exciting drug strategy for targeting HIV-1. Inhibitors that prevent complex formation would be potential candidates for HIV-1 suppression, and purification of these Vif complexes in homogeneous form would provide the basis for screens to identify and evaluate inhibitor candidates. Thus, our strategy for purifying Vif-Cul5-CBFβ-EloB/C complexes may lead to useful screening approaches for identifying novel anti-HIV drug candidates.
